# The Pel polysaccharide is predominantly composed of a dimeric repeat of α-1,4 linked galactosamine and *N-*acetylgalactosamine

**DOI:** 10.1038/s42003-022-03453-2

**Published:** 2022-05-26

**Authors:** François Le Mauff, Erum Razvi, Courtney Reichhardt, Piyanka Sivarajah, Matthew R. Parsek, P. Lynne Howell, Donald C. Sheppard

**Affiliations:** 1 Department of Microbiology and Immunology, Faculty of Medicine, McGill University, Montreal, QC Canada; 2 Infectious Disease in Global Health Program, McGill University Health Centre, Montreal, QC Canada; 3 McGill Interdisciplinary Initiative in Infection and Immunity, Montreal, QC Canada; 4 Program in Molecular Medicine, Research Institute The Hospital for Sick Children, Toronto, ON Canada; 5 Department of Biochemistry, University of Toronto, Toronto, ON Canada; 6 Department of Microbiology, University of Washington, Seattle, WA USA; 7 Present Address: Department of Chemistry, Washington University, St. Louis, MO 63130 USA

**Keywords:** Biofilms, Bacteria

## Abstract

The genetic capacity to synthesize the biofilm matrix exopolysaccharide Pel is widespread among Gram-negative and Gram-positive bacteria. However, its exact chemical structure has been challenging to determine. Using a *Pseudomonas aeruginosa* strain engineered to overproduce Pel, improvements to the isolation procedure, and selective hydrolysis with the glycoside hydrolase PelA_h_, we demonstrate that Pel is a partially de-*N-*acetylated linear polymer of α-1,4-*N-*acetylgalactosamine comprised predominantly of dimeric repeats of galactosamine and *N-*acetylgalactosamine.

## Abstract (web summary)

The structure of the Pel exopolysaccharide from Pseudomonas aeruginosa is fully characterized.

## Introduction

Biofilms are aggregates of microorganisms attached to each other and/or to a surface encased in a self-produced extracellular matrix^1^. This matrix, composed of proteins, extracellular DNA, lipids, and polysaccharides, creates a unique microenvironment, and enables the biofilm community to have emergent properties that are different from planktonic bacteria^2^. Exopolysaccharides are vital to the biofilm matrix and contribute to adhesion, cell-to-cell interactions, and protection from antimicrobials and host immune responses^3^. Exopolysaccharides produced by microorganisms are structurally very diverse with linear and branched homo- and hetero-polymers composed of distinct monosaccharides that are produced by unique biosynthetic pathways. These polymers can also be further modified post-polymerization through the action of various carbohydrate-active enzymes (CAZymes)^4^.

*Pseudomonas aeruginosa* is a Gram-negative bacterium that has been the focus of intense research due to its prominent role in disease and ability to form biofilms on medical devices and human tissues^5,6^. *P. aeruginosa* is recognized as a primary pathogen for individuals with cystic fibrosis (CF), who become chronically infected with the bacterium. The failure to clear the infection from CF airways leads to inflammation and lung disease, and *P. aeruginosa* remains one of the leading causes of morbidity and mortality in this patient population^6^. *P. aeruginosa* is genetically capable of producing at least three distinct exopolysaccharides; alginate, Psl, and Pel^7^. Alginate is primarily associated with chronic infections and used for biofilm formation by mucoid *P. aeruginosa* strains^8^. Aggregates of non-mucoid clinical isolates in CF airways have recently been shown to express both Pel and Psl^9^.

Pel-dependent biofilm formation in *P. aeruginosa* requires the activity of a seven gene operon, *pelABCDEFG*, whose products mediate sugar polymerization and transport across the cytoplasm (PelDEFG), modification by hydrolase and deacetylase activities (PelA), and export (PelBC) of the polymer into the extracellular milieu^10–14^. We recently identified a variant form of this gene cluster, *pelDEA*_*DA*_*FG*, in numerous Gram-positive bacterial species and have shown that this locus is required for biofilm formation in *Bacillus cereus* ATCC 10987^15,16^. Our bioinformatics analyses have now identified that more than 1400 Gram-positive and Gram-negative bacterial species have the genetic capacity to synthesize Pel, making the Pel biosynthetic locus one of the most phylogenetically widespread biofilm matrix determinants in bacteria^17^. Determination of the role of Pel in the formation, maintenance, and properties of biofilms by this wide range of organisms has been hampered by our incomplete knowledge of its chemical structure.

An engineered Pel overexpression strain, PAO1 Δ*wspF* Δ*psl* P_BAD_*pel* (P_BAD_*pel*), has been used extensively in the literature to study Pel. In this strain a polar mutation in the *psl* operon and the replacement of the native promotor region of *pelA* with the araC-P_BAD_ promoter on the PAO1 chromosome allows for the arabinose-dependent expression of the *pel* operon specifically^12^. The in-frame mutation in *wspF*, a negative regulator of the diguanylate cyclase WspR, results in a high bis-(3′-5′)-cyclic dimeric guanosine monophosphate (c-di-GMP) background which transcriptionally and post-translationally activates Pel biosynthesis^12,18,19^. P_BAD_*pel* allows for robust, inducible expression of Pel and was used to show that the Pel polymer is de-*N*-acetylated, conferring a cationic charge at physiological pH that facilitates interactions with anionic host polymers such as DNA, and increases antimicrobial tolerance^9,20^. Subsequent studies using P_BAD_*pel* have identified the *Wisteria floribunda* (WFL) lectin as being Pel specific, suggesting that the polymer contains terminal *N-*acetylgalactosamine (GalNAc) moieties^20^. Previous glycosyl composition, linkage analysis, and solid-state NMR of Pel from P_BAD_*pel* have reported that secreted Pel is a cationic polymer of 50% de-*N*-acetylated 1–4 linked GalNAc and *N*-acetylglucosamine (GlcNAc) in a 5:1 ratio^9,20^.

Attempts to fully characterize the anomeric configuration and structure of Pel have been hampered in the past by the insolubility of the polymer. Using a modified isolation procedure and hydrolysis with PelA_h_, the recombinantly expressed glycoside hydrolase domain of the Pel modification enzyme, PelA^21^, we overcame these challenges and herein present the chemical structure of Pel. We determined that Pel does not contain GlcNAc and is a linear homopolymer of partially de-*N-*acetylated α-1,4-GalNAc comprised predominantly of dimeric repeats of galactosamine (GalN) and GalNAc.

## Results and discussion

### Pel is a polymer of partially de-N-acetylated 1,4 linked N-acetylgalactosamine

**Fig. 1:**
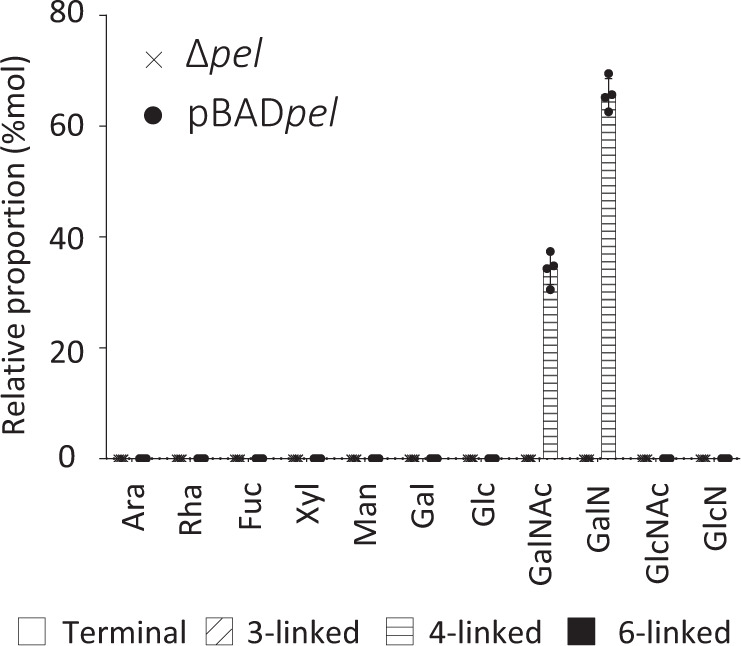
Pel is a partially de-*N*-acetylated polymer of 1,4-*N*-acetylgalactosamine. Monosaccharide composition and linkage analysis of Pel by GC-MS. Average and standard deviation of four biological replicates are represented. Histogram patterns represent the linkage found in the PMAA derivatization (no pattern: terminal residues, diagonal dash: 3-linked residues, horizontal dash: 4-linked residues, black filled: 6-linked residues). Ara arabinose, Rha rhamnose, Fuc fucose, Xyl xylose, Man mannose, Gal galactose, Glc glucose, GalNAc *N*-acetylgalactosamine, GalN galactosamine, GlcN glucosamine, GlcNAc *N*-acetylglucosamine.

Secreted Pel was isolated from the supernatants of the genetically engineered P_BAD_*pel* strain and the isogenic Pel-deficient PAO1 Δ*wspF* Δ*psl* Δ*pel* (Δ*pel*) strain as a negative control. The monosaccharide composition of Pel was analyzed via gas chromatography coupled to mass spectrometry (GC-MS) following reductive amination of the polymer hexosamines, polymer hydrolysis, re-*N*-acetylation and trimethylsilyl derivatization of the monosaccharides. GalN was found to be the main monosaccharide present in Pel with an average relative abundance of 66 ± 2.9% (Fig. 1). The rest of the sample was composed of GalNAc (33 ± 2.9%, Fig. 1). Partially methylated alditol acetate derivation of chemically re-*N*-acetylated Pel was used to determine the linkages between GalNAc/GalN residues of secreted Pel. Only 4-linked GalNAc and GalN residues were found. (Fig. 1). Unlike previous studies^20^, GlcNAc was not detected in our GC-MS analyses. We hypothesize that the 4-GlcNAc previously reported in secreted Pel likely originated from contaminating peptidoglycan^22^, fragments of which may have been released into the culture supernatant following cell lysis or outer membrane vesicle release^23,24^. Consistent with this hypothesis, the previous analysis of Pel composition also reported the presence of GlcNAc and high amounts of glucose in the secreted polysaccharide isolated from the Pel-deficient Δ*pel* strain^20^. These monosaccharides were not detected in our analyses of the Pel-deficient Δ*pel* strain (Fig. 1), suggesting that the incorporation of an extensive dialysis step was effective at eliminating these contaminating sugars. Taken as a whole, these GC-MS analyses reveal that secreted Pel is a linear polymer of partially de-*N*-acetylated 1,4 linked GalNAc.

### Pel is a polysaccharide composed of α-linked monosaccharides

**Fig. 2:**
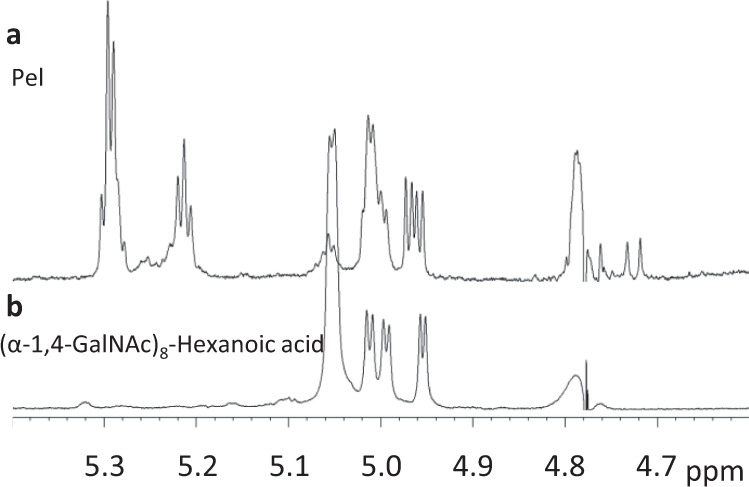
Pel is composed of α-anomer monosaccharides. **a** ^1^H NMR spectrum of re-*N*-acetylated Pel digested by PelA_h_. Summary of signals of interest: δ 4.72 (d, J = 8.14 Hz), 4.96 (d, J = 3.97 Hz), 4.97 (d, J = 3.97 Hz), 4.99 (d, J = 3.41 Hz), 5.01 (d, J = 2.70 Hz), 5.05 (d, J = 3.36 Hz), 5.29 (d, J = 3.88 Hz). **b** ^1^H NOESY NMR profile of synthetic (α-1,4-GalNAc)_8_. Summary of signals of interest: 4.95 (d, J = 3.83 Hz), 4.99 (d, J = 3.87 Hz), 5.01 (d, J = 3.94 Hz), 5.05 (d, J = 3.35 Hz).

The determination of the anomeric configuration of the monosaccharides in Pel was performed using ^1^H NMR spectroscopy. Prior to the spectral acquisition, solubilization of Pel was achieved through re-*N*-acetylation and enzymatic hydrolysis of the re-*N*-acetylated Pel with the recombinantly expressed hydrolase domain of PelA (PelA_h_). This treatment released short soluble oligosaccharides of 1,4-GalNAc^25^. Previous work from our group has shown that PelA_h_ can cleave 1,4-GalNAc oligosaccharides composed of 7 or more GalNac, releasing products as small as trimers^25^. The resulting ^1^H NMR spectrum (Fig. 2a) was compared to that of a synthetic α-1,4-linked homo-GalNAc octasaccharide with 6-hydroxy-hexanoic acid coupled to its reducing end (Fig. 2b)^26^. The spectrum of the synthetic oligosaccharide revealed four doublets at 4.95 ppm (J = 3.8 Hz), 4.99 ppm (J = 3.9 Hz), 5.01 ppm (J = 3.9 Hz), and 5.05 ppm (d, J = 3.4 Hz) (Fig. 2b). With the exception of the doublet at 5.05 ppm, all the doublets displayed a coupling constant between 3.8 and 3.9 Hz characteristic of monosaccharides with an α-stereochemistry (Fig. 2b). An almost identical set of four doublets was found within the Pel ^1^H NMR spectrum (Fig. 2a) suggesting that the polysaccharide is composed of α-GalNAc and α-GalN.

Four additional signals were observed in the Pel spectrum at 4.72, 4.97, 5.23, and 5.30 ppm (Fig. 2a). These signals are likely attributable to artefacts arising from the chemical and enzymatic reactions performed to overcome Pel insolubility. Among these signals, only two could be matching with potential protons from a monosaccharide anomeric carbon, the signals at 4.72 ppm (d, J = 8.1 Hz) and 4.97 ppm (d, J = 4.0 Hz). The doublet at 4.72 ppm has a coupling constant indicative of a β-anomer configuration and is likely a consequence of polymer cleavage by PelA_h_ as was previously shown in an analysis of PelA_h_-digested oligosaccharides^25^. As the newly released reducing ends are free to mutarotate, they produce two doublets at 4.7 ppm and at 5.3 ppm originating from the β and α-anomer, respectively^25^. In the Pel spectra, a signal at 5.3 ppm was also observed (Fig. 2a) and could match with an α-anomer present at the reducing end of the oligosaccharide. However, the abnormally high intensity and multiplicity of this signal may indicate the presence of a second signal of unknown origin at this chemical shift (Fig. 2a). Signals corresponding to mutarotation at the reducing end of the synthetic (α-1,4-GalNAc)_8_-hexanoic acid were not observed as mutarotation was prevented by the presence of the aglycone (Fig. 2b). Finally, we attribute the doublet at 4.97 ppm in the Pel spectrum to residual GalN present within Pel (Fig. 2a). Prior to the enzyme digestion, Pel was chemically re-*N*-acetylated to enhance PelA_h_ activity^25^. However, as Pel was insoluble at this step, we hypothesize that acetylation may have been incomplete and that GalN-containing oligosaccharides were also likely released by PelA_h_ treatment.

Taken together, the high similarity between the spectra of the synthetic oligosaccharide and soluble Pel oligosaccharides, as well as the absence of significant signal indicative of beta-stereochemistry within the polymer spectra, suggests that Pel is composed of monosaccharides in the α-configuration.

### Pel is predominantly constituted of a dimer repeat (GalN-GalNAc)

**Fig. 3:**
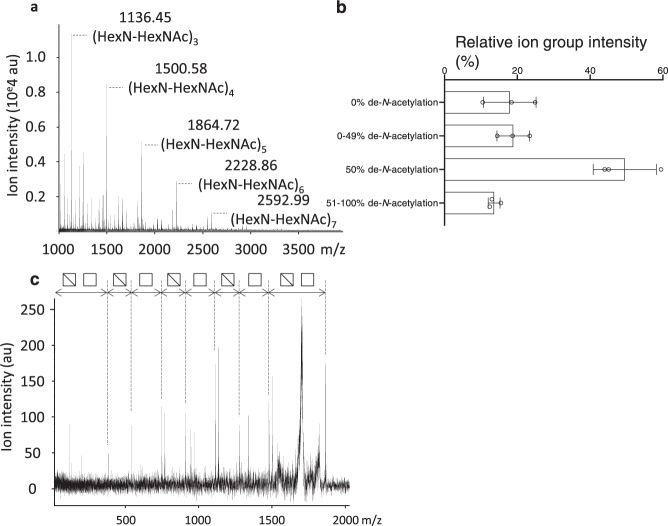
Pel is predominantly composed of a repeating GalN-GalNAc dimers. **a** MALDI-TOF MS spectra of Pel after partial acid hydrolysis. HexNAc *N*-acetylhexosamine (white squares), HexN hexosamine (cross white squares). **b** Relative ion composition of Pel after partial acid hydrolysis. Ion groups were categorized by de-*N*-acetylation status. **c** MSMS fragmentation profile of the ion at *m/z* 1864.72 corresponding to (HexN-HexNAc)_5_.

To study Pel monosaccharide arrangement within the polymer, Pel was partially hydrolyzed with hydrochloric acid. The analysis of the released oligosaccharides by MALDI-TOF mass spectrometry revealed that ~50% of the oligosaccharide population was comprised of an equal number of hexosamine (HexN) and *N*-acetylhexosamine (HexNAc) residues (Fig. 3a and b). MSMS fragmentation of these ions revealed that the oligosaccharides were composed of a dimeric repeat of HexN-HexNAc (Fig. 3c). The remaining 50% of ions found in the spectra were identified as homo-α-1,4-HexNAc with different degrees of de-*N*-acetylation (Fig. 3b). No de-*N*-acetylation was observed for ~18% of the ions, partial de-*N*-acetylation (less than 50%) was observed for ~19% of the ions, and about 14% of the ions exhibited more than 50% de-*N*-acetylation (Fig. 3b). Combined, these data indicate that Pel is comprised predominantly of GalN-GalNAc repeating units.

### Enzymatic confirmation of Pel structure

**Fig. 4:**
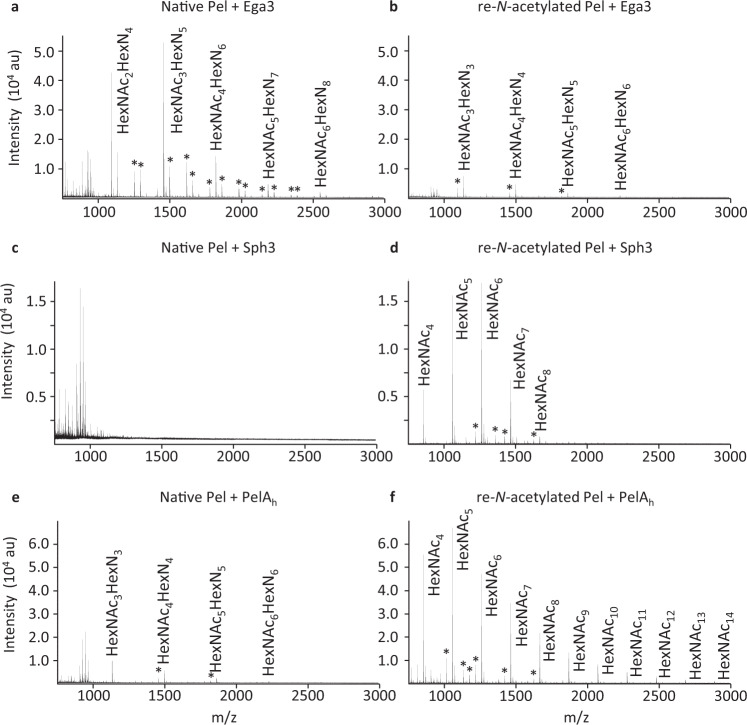
Activity of enzymes involved in the hydrolysis of α-(1-4)-galactosamine and α-(1-4)-*N*-acetylgalactosamine polymers. MALDI-TOF enzyme fingerprinting of Pel performed using either native Pel (panels **a**, **c**, **e**) or chemically re-*N*-acetylated Pel as a substrate (panels **b**, **d**, **f**). **a**, **b** Ega3 fingerprints. **c**, **d** Sph3 and **e**, **f** PelA_h_ fingerprints. Asterisk (*) indicates non-annotated ion of known composition. Digestions were performed three times; a representative spectrum is displayed for each condition.

**Fig. 5:**
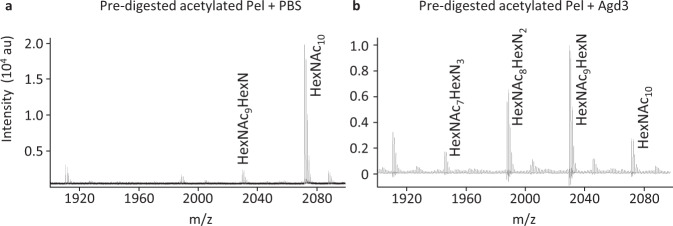
Activity of Agd3, an enzyme involved in the de-*N*-acetylation of α-(1-4)-*N*-acetylgalactosamine polymers. MALDI-TOF enzyme fingerprinting of **a** PelA_h_ pre-digested Pel used as substrate and incubated with PBS. **b** Agd3 degradation of the PelA_h_ pre-digested Pel.

To further validate our findings, we used MALDI-TOF enzyme fingerprinting with a panel of recently characterized α-1,4-GalNAc specific CAZymes (Figs. 4 and 5, Supplementary Fig. 1). Using native or re-*N-*acetylated Pel as substrates, we found that the endo α-1,4-galactosaminidase Ega3 cleaved native Pel, producing a specific HexN_2_ MS fingerprint at the reducing end of dimer repeats of HexN-HexNAc^27^ (Fig. 4a). As anticipated, the re-*N-*acetylated polymer was more resistant to Ega3 degradation (Fig. 4b). Conversely, while the endo-α-1,4-*N*-acetylgalactosaminidases, Sph3 and PelA_h_, both degraded re-*N-*acetylated Pel, only PelA_h_ was able to release small amounts of GalN-GalNAc oligosaccharides from the native polymer (Fig. 4c-f). This is consistent with previous findings revealing that despite sharing the same enzymatic activity, differences in active site architecture result in Sph3 being specific for poly-GalNAc, while PelA_h_ is able to tolerate the presence of GalN within the target polymer^25^. No oligosaccharide products were detected in native Pel and re-*N*-acetylated Pel samples incubated in PBS, confirming that the oligosaccharides detected by MALDI-TOF were the product of the action of the respective CAZyme (Supplemental Fig. 2).

Lastly, Agd3, an α-(1-4)-*N*-acetylgalactosamine deacetylase was able to de-*N-*acetylate re-*N-*acetylated Pel (Fig. 5)^28^. Collectively, these enzymatic studies add further evidence that Pel is a partially de-*N*-acetylated polymer of α-1,4-GalNAc.

## Discussion

Although secreted *P. aeruginosa* Pel has previously been characterized as a cationic polymer of 50% de-*N*-acetylated 1-4 linked GalNAc and GlcNAc in a 5:1 ratio^9,20^ determination of the anomeric configuration and structure of Pel have been hindered by its insolubility. Using P_BAD_*pel*, a *P. aeruginosa* strain engineered to overproduce Pel, improvements to the isolation procedure, recombinantly expressed PelA_h_, and a combination of MS and NMR analyses, we have determined that Pel is a linear homopolymer of partially de-*N-*acetylated α-1,4-linked GalNAc that is comprised predominantly of dimeric repeats of GalN and GalNAc. Notably, we found that Pel does not contain GlcNAc^20^.

The P_BAD_*pel* strain was used to determine the structure of Pel due to its ability to produce sufficient quantities of the polymer for structural studies. This strain has been used to characterize Pel-dependent phenotypes and has been extensively compared to *P. aeruginosa* PA14, an intrinsically Pel-producing strain in which the *pel* operon was the first identified^10^. Both P_BAD_*pel* and PA14 require PelA deacetylase activity for Pel-dependent biofilm formation, have the same phenotypes in crystal violet assays measuring biofilm adherence, and wrinkly colony morphologies on Congo red plates indicative of exopolysaccharide production^12^. P_BAD_*pel* and PA14 both form flow cell biofilms that have signals with fluorescein-labeled WFL^20,29^. In addition, biofilms formed by P_BAD_*pel* and PA14 can be disrupted by the exogenous addition of PelA_h_, an endo-α-1,4-*N*-acetylgalactosaminidase with a deep electronegative active site able to bind and cleave cationic partially de-*N*-acetylated GalNAc oligosaccharides^25,30^. Given the corresponding phenotypes, we anticipate the chemical composition of secreted Pel produced by P_BAD_*pel* and PA14 will be equivalent.

Knowledge of the chemistry and structure of Pel provides more insight and substantiates previous findings regarding its biosynthesis. For example, the lack of GlcNAc in Pel correlates with the absence of multiple glycosyltransferases and an epimerase in the *P. aeruginosa pel* operon^10,16^. Multiple glycosyltransferases encoded in an operon frequently indicate that more than one type of monosaccharide is incorporated into the polymer, as seen in the *psl* operon, which contains the glycosyltransferases *pslC, pslH*, and *pslI*, and produces a repeating branched pentasaccharide containing D-mannose, L-rhamnose, and D-glucose^7,31^. In the *P. aeruginosa alg* operon, a C5-epimerase, AlgG, converts D-mannuronate (D-ManA) to L-guluronate (L-GulA), producing bacterial alginate, a randomly acetylated linear polysaccharide composed of β-1,4-linked D-ManA and varying amounts of L-GulA^4,32,33^. The presence of only one glycosyltransferase and the absence of an epimerase to perform post-polymerization modification in the *P. aeruginosa pel* operon had long suggested that Pel was a homopolymer, which the data presented herein confirms.

Similarly, our finding that Pel does not contain GlcNAc also adds support to the proposal that the key sugar-nucleotide precursor involved in Pel biosynthesis is likely, GalNAc bound to uridine 5′-diphosphate (UDP)^34^. Previous studies have already established that PelF, the putative Pel glycosyltransferase, binds UDP^20^. The genes encoding enzymes for precursor generation in *P. aeruginosa* for Psl and alginate are found within and/or adjacent to their associated exopolysaccharide gene clusters. However, in *P. aeruginosa*, the *pel* operon does not contain genes that encode an enzyme capable of producing the sugar-nucleotide precursor, indicating that they have to be encoded elsewhere on the chromosome^7,10,12,34^. We have previously identified PelX, a UDP-GlcNAc C4-epimerase in *Pseudomonas protegens* Pf-5 that generates UDP-GalNAc precursors^34^. PelX and its paralogue, PgnE, were found to be critical for Pel polysaccharide production in this species. While, an active UDP-GlcNAc C4-epimerase has not been identified in *P. aeruginosa* Pel biosynthesis, it is likely that PA4068, which shares 76% identity with PgnE, fulfils this role^34^. Confirmation that the Pel polysaccharide is a homopolymer of de-*N*-acetylated GalNAc supports the requirement for an active UDP-GlcNAc C4-epimerase to generate UDP-GalNAc, thus warranting further investigation of the role of PA4068 in *P. aeruginosa* Pel biosynthesis.

The identification of the Pel structure reveals its high degree of similarity to the fungal *Aspergillus fumigatus* biofilm exopolysaccharide, galactosaminogalactan (GAG), a heteropolysaccharide composed of α-1,4 linked Gal, GalNAc, and GalN^35–37^. Both GAG and Pel have an α-anomeric configuration, are 1,4 linked, and contain GalNAc and GalN, thus explaining the cross-kingdom activity of the fungal and bacterial glycoside hydrolases, Ega3 and PelA_h_, to disrupt both *A. fumigatus* GAG and *P. aeruginosa* Pel biofilms^27,30^. NMR and molecular dynamics simulations performed on synthetic (α-1,4-GalNAc)_7_ to investigate the conformation and spatial presentation of the oligomer revealed that C2 acetyl substituents are on alternating sides of the longitudinal axis of the polymer. These data suggest that the dimeric GalN-GalNAc repeat present in the Pel structure, is the result of PelA de-*N-*acetylating one face of the polymer processively^26^. Knowledge of the Pel polysaccharide composition coupled with a structure of PelA will aid in understanding the mode of action of this CAZyme.

Our recent bioinformatics analyses found the *pel* operon to be widespread in a diverse array of eubacteria^15,17^, suggesting that partially de-*N*-acetylated α-1,4 linked GalNAc exopolysaccharides are probably not limited to *Pseudomonas* spp*, Bacillus cereus*, and *A. fumigatus* biofilms^16,20,37^. The extraordinary conservation of the *pel* operon across organisms suggests that other bacterial species also secrete a partially de-*N*-acetylated α-1,4-GalNAc exopolysaccharide and that Pel could play a greater role in virulence than previously appreciated. Targeting this polymer at the post-transcriptional, post-translational, or post-polymerization level could represent a promising avenue for diagnostic and personalized antimicrobial treatments based on the exopolysaccharide composition determined in this work.

## Methods

### Polysaccharide isolation

The Pel overexpression strain PAO1 Δ*wspF* Δ*psl* P_BAD_ *pel* (P_**BAD**_*pel*) and negative control strain PAO1 Δ*wspF* Δ*psl* Δ*pel* (Δ*pel*) were cultured and Pel isolated as described previously^20^ with the following modifications: (i) Jensen’s medium was inoculated with 2 mL of overnight culture; (ii) after the culture supernatant precipitate was washed three times with 95–100% (v/v) ethanol, it was left to air dry before resuspension in 20 mL of buffer (1 mM CaCl_2_, 2 mM MgCl_2_ in 50 mM Tris, pH 7.5); (iii) the resuspended precipitate was treated with 15 mg DNase I and 15 mg RNaseA overnight at 37 °C, followed by 20 mg proteinase K overnight at 37 °C; (iv) after heating the enzyme-treated sample at 95 °C for 10 min it was centrifuged at 36,000 × *g* for 10 min at 20 °C; and (v) in the final step the sample was extensively dialyzed against water using 50 kDa molecular weight cut tubing prior to being flash-frozen and lyophilized.

### De-N-acetylation quantification by reductive amination and monosaccharide composition by Gas chromatography coupled to mass spectrometry (GC-MS)

To perform reductive amination, samples were reconstituted in a solution of 1 M sodium cyanoborohydride reconstituted in dimethylsulfoxide (DMSO): acetic acid (70:30% v/v) and 50% acetone in a 1:1 ratio. After 16 h at 60 °C, samples were derivatized with Trimethylsilyl as reported previously^38^. Briefly, samples were hydrolyzed with either 2 M trifluoroacetic acid (TFA) for 2 h at 110 °C, or 6 M hydrochloric acid (HCl) for 4 h at 100 °C to quantify non-amino and amino monosaccharides, respectively, and then methanolysed with 1 M HCl in methanol overnight at 80 °C. Hexosamine residues were re-*N*-acetylated using a solution of methanol: pyridine: acetic anhydride (10:2:3) for 1 h at room temperature. Residues were then silylated with a mix of hexamethyldisiloxane: trimethylchlorosilane: pyridine (3:1:9). Quantification of reduced hexosamines to *N*-acetylhexosamines was performed by using GC-MS by injecting the TMS derivates into the Agilent Technology Ensemble 5977B GC-MS equipped with a CP-Sil5CB capillary column using a temperature gradient as previously reported^25^.

### Pel chemical re-N-acetylation

Purified Pel samples were incubated in a solution of methanol: pyridine: acetic anhydride (10:2:3) overnight at room temperature, and then washed twice with pure methanol.

### Linkage analysis

Partially methylated alditol acetate (PMAA) derivatives of re-*N-*acetylated Pel were made using a modified protocol^38^. Briefly, samples were first permethylated with 500 µL of iodomethane in a slurry of DMSO: sodium hydroxide for 2 h at room temperature. Permethylated polymers were hydrolyzed for 4 h in 2 M TFA. Released monosaccharides were reduced with 10 mg/mL sodium borodeuteride in 1 M ammonium hydroxide overnight and then acetylated by incubation in pyridine: acetic anhydride (1:1) for 1 h at 100 °C. Analysis of the PMAA derivatives was carried out by GC-MS as reported above. The 4-GalNAc linkage was identified by comparing the chromatogram to one obtained from *Aspergillus* galactosaminogalactan.

### Anomer determination by nuclear magnetic resonance

Proton NMR spectra were recorded on an AVANCE III HD 600 NMR spectrometer (Ascend™ 600 magnet - Bruker Biospin Ltd.) operating at a frequency of 600.17 MHz for ^1^H and equipped with a quadruple resonance CryoProbe (CPQCI 1H-31P/13 C/15 N) and a SampleJETTM autosampler. 1.5 mg of re-*N*-acetylated Pel was incubated with 20 µM of PelA_h_ in 1X PBS in D_2_O for 60 h and transferred into a 3 mm NMR tube containing trimethylsilylpropanoic acid at a final concentration of 0.5 mM. Synthetic (α-1,4-GalNAc)_8_-hexanoic acid, generously provided by Dr. Codée (Leiden University) served as a control. The ^1^H spectra were acquired using the pulse sequence *noesypr1d* (Nuclear Overhauser Effect Spectroscopy 1D, Bruker Biospin Ltd) in order to achieve good suppression of the water signal. ^1^H spectrum was acquired with 64 scans, a ^1^H 90° pulse length of 7.8 μs, a mixing time of 10 ms, a spectral width of 12 kHz, and a recycle delay of 4 s for a total of 66 K data points. Anomer configuration was confirmed by 2D ^1^H–^1^H Correlation Spectroscopy (COSY) spectra at 25 °C with spectra acquired using 16 scans with a ^1^H 90° pulse length of 8 μs, a spectral width of 3 kHz in both dimensions, a repetition delay of 1.8 s for a total of 2048 data points in F2 and 128 increments in F1. All spectra were processed using TOPSPIN software (version 3.5 pl 7, Bruker Biospin Ltd).

### Partial acid hydrolysis for Matrix-Assisted Laser Desorption/Ionization-Time of Flight (MALDI-TOF) analysis

One milligrams of Pel was hydrolyzed by 0.1 M HCl for 2 h at 100 °C. Hydrolysates were neutralized by sodium hydroxide and released oligosaccharides were reduced with sodium borodeuteride at 10 mg/mL in 1 M ammonium hydroxide overnight. Reduced oligosaccharides were then purified using a HyperSep HyperCarb column (Chromatography Specialist) as previously described^39^_._ Briefly, after conditioning the column with 5 mL of acetonitrile (ACN) and 5 mL of water, oligosaccharides were loaded on the column and washed with 5 mL of water and 5 mL of 5% (v/v) ACN. Elution was performed using 2 mL of 50% (v/v) ACN. Samples were concentrated and reconstituted in 10 µL of 0.2% (v/v) TFA and spotted in 2,5-dihydroxybenzoic acid (DHB) matrix at 5 mg/mL in ACN:0.2% TFA (70:30%, v/v). Spectra were recorded on a Bruker UltrafleXtreme in positive reflector mode and represent an accumulation of 10,000 laser shots. MALDI-TOF and tandem mass spectrometry (MSMS) experiments were performed using the same mass spectrometer.

### Enzymatic MS fingerprinting

The following recombinant proteins used for MS fingerprinting were expressed and purified as previously described: PelA_h_^25^, Ega3^27^, Sph3^25^, and Agd3^28^. Digestions with 1 µM Ega3, Sph3, or PelA_h_ were carried out for 1 h in 1 × PBS with 1 mg/mL of native Pel, or re-*N*-acetylated Pel. Deacetylation by 1 µM Agd3 used re-*N*-acetylated Pel digested with PelA_h_ as the substrate. The released oligosaccharides were purified using a HyperSep HyperCarb column (Chromatography Specialist) as described above. Enzymatic MALDI-TOF fingerprints were acquired as described above.

### Statistics and reproducibility

At least three biological replicates of Pel extracts were analyzed for all the experiments.

### Reporting summary

Further information on research design is available in the Nature Research Reporting Summary linked to this article.

## Supplementary information

Peer Review File

Supplementary Information

Reporting Summary

## Data Availability

The authors declare that all data supporting the findings of this study are available within the paper and its supplementary information files. Raw dataset for Figs. 1 and 3b can be found on https://figshare.com/account/home#/projects/135887.
