# Author Correction: The Pel polysaccharide is predominantly composed of a dimeric repeat of α-1,4 linked galactosamine and *N*-acetylgalactosamine

**DOI:** 10.1038/s42003-022-03567-7

**Published:** 2022-06-24

**Authors:** François Le Mauff, Erum Razvi, Courtney Reichhardt, Piyanka Sivarajah, Matthew R. Parsek, P. Lynne Howell, Donald C. Sheppard

**Affiliations:** 1 Department of Microbiology and Immunology, Faculty of Medicine, McGill University, Montreal, QC Canada; 2 Infectious Disease in Global Health Program, McGill University Health Centre, Montreal, QC Canada; 3 McGill Interdisciplinary Initiative in Infection and Immunity, Montreal, QC Canada; 4 Program in Molecular Medicine, Research Institute The Hospital for Sick Children, Toronto, ON Canada; 5 Department of Biochemistry, University of Toronto, Toronto, ON Canada; 6 Department of Microbiology, University of Washington, Seattle, WA USA; 7 Present Address: Department of Chemistry, Washington University, St. Louis, MO 63130 USA

**Keywords:** Biofilms, Bacteria

Correction to: *Communications Biology* 10.1038/s42003-022-03453-2, published online 26 May 2022.

The original version of the Article contained an error in Figure 1, where the X-axis labels GalNAc and GalN were inadvertently swapped and the labels should read Δpel and pBADpel. The correct version of Figure 1 is:
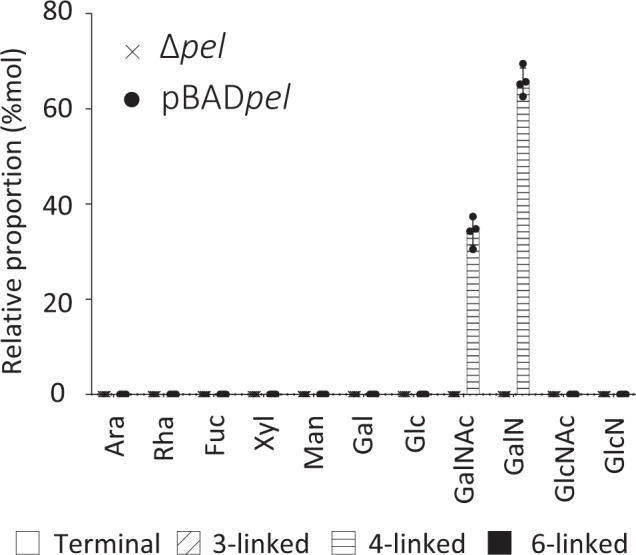


which replaces the previous incorrect version
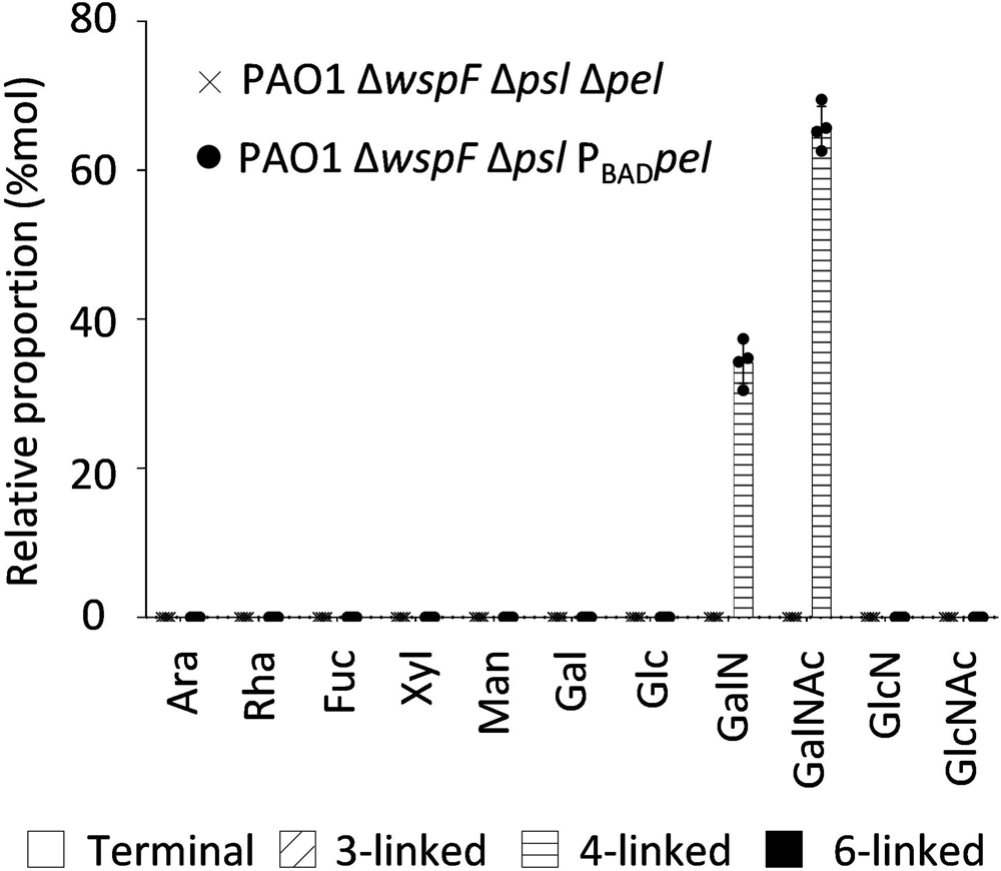


Accordingly, the Figure Legend for Figure 1 previously stated: “Fig. 1 Pel is a partially de-N-acetylated polymer of 1,4-Nacetylgalactosamine. Monosaccharide composition and linkage analysis of Pel by GC-MS. Average and standard deviation of four biological replicates are represented. Histogram patterns represent the linkage found in the PMAA derivatization (no pattern: terminal residues, diagonal dash: 3-linked residues, horizontal dash: 4-linked residues, black filled: 6-linked residues). Ara arabinose, Rha rhamnose, Fuc fucose, Xyl xylose, Man mannose, Gal galactose, Glc glucose, GalN galactosamine, GalNAc *N*-acetylgalactosamine, GlcN glucosamine, GlcNAc *N*-acetylglucosamine”.

The Figure Legend should read: “Fig. 1 Pel is a partially de-N-acetylated polymer of 1,4-Nacetylgalactosamine. Monosaccharide composition and linkage analysis of Pel by GC-MS. Average and standard deviation of four biological replicates are represented. Histogram patterns represent the linkage found in the PMAA derivatization (no pattern: terminal residues, diagonal dash: 3-linked residues, horizontal dash: 4-linked residues, black filled: 6-linked residues). Ara arabinose, Rha rhamnose, Fuc fucose, Xyl xylose, Man mannose, Gal galactose, Glc glucose, GalNAc *N*-acetylgalactosamine, GalN galactosamine, GlcN glucosamine, GlcNAc *N*-acetylglucosamine”.

Further, in the original version of the Article, the third paragraph of the Discussion incorrectly stated “Multiple glycosyltransferases encoded in an operon frequently indicate that more than one type of monosaccharide is incorporated into the polymer, as seen in the psl operon, which contains the glycosyltransferases pslF, pslH, and pslI, and produces a repeating branched pentasaccharide containing d-mannose, l-rhamnose, and d-glucose^7,31^”.

The text should read “Multiple glycosyltransferases encoded in an operon frequently indicate that more than one type of monosaccharide is incorporated into the polymer, as seen in the psl operon, which contains the glycosyltransferases pslC, pslF, pslH, and pslI, and produces a repeating branched pentasaccharide containing d-mannose, l-rhamnose, and d-glucose^7,31^”.

The errors have been corrected in both the PDF and HTML versions of the Article.

