# Supplementary material for: The Pel polysaccharide is predominantly composed of a dimeric repeat of α-1,4 linked galactosamine and N-acetylgalactosamine
Source: Commun Biol. 2022 May 26;5:502. doi: 10.1038/s42003-022-03453-2 (PMC9135694; doi:10.1038/s42003-022-03453-2)
Supplement: Supplementary file 2 — Supplementary Information [file 42003_2022_3453_MOESM2_ESM.pdf]

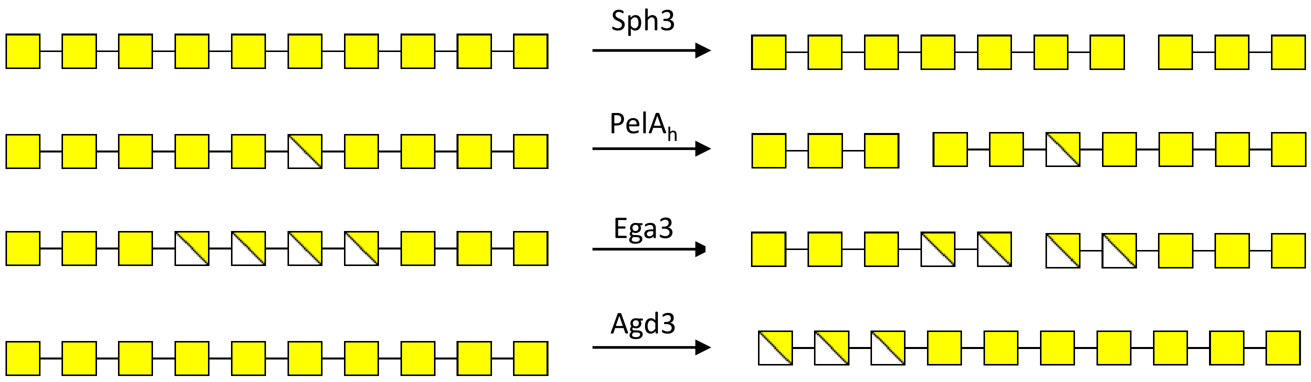

Supplemental Figure 1. Schematic of CAZyme functions on fully or partially de-*N*-acetylated α-(1-4)-*N*-acetylgalactosamine polymers. Sph3 is a strict endo-1,4-*N*-acetylgalactosaminidase. PelA is endo-1,4-*N*-acetylgalactosaminidase able to tolerate the presence of GalN within the binding site. Ega3 is an endo-1,4-galactosaminidase and Agd3 is an α-(1-4)-*N*-acetylgalactosamine deacetylase. Yellow squares represent GalNAc. Half yellow squares represent GalN.

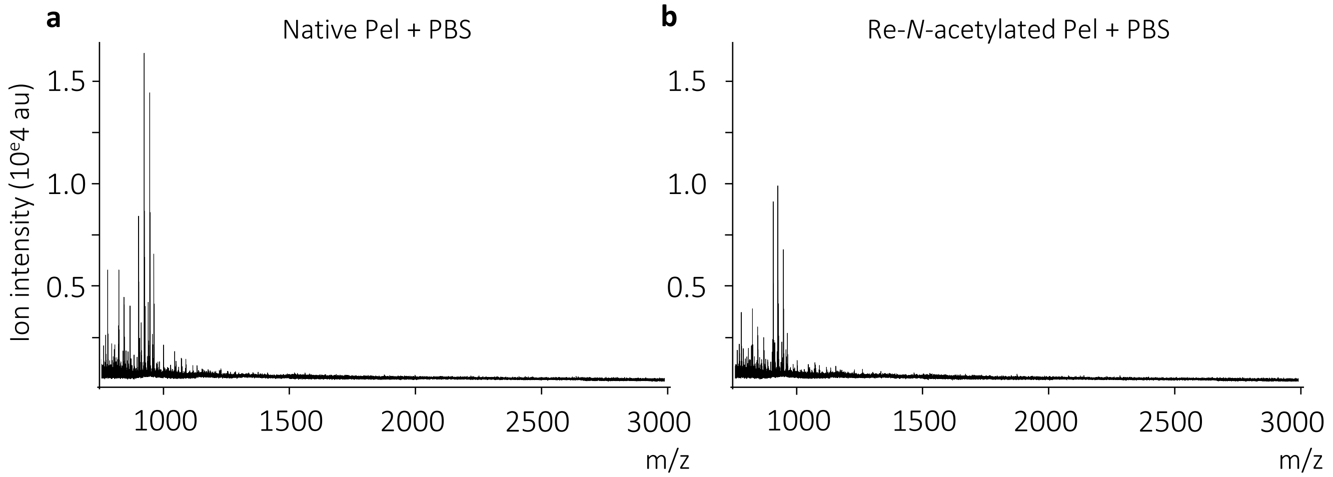

Supplemental Figure 2: Oligosaccharides detected by MALDI-TOF enzyme fingerprinting are specifically a result of CAZyme activity on isolated Pel. **a.** Native Pel incubated with PBS. **b.** Re-*N*-acetylated Pel incubated with PBS. All ions observed are from the DHB matrix.
