# Supplementary material for: The Pel polysaccharide is predominantly composed of a dimeric repeat of α-1,4 linked galactosamine and N-acetylgalactosamine
Source: Commun Biol. 2022 May 26;5:502. doi: 10.1038/s42003-022-03453-2 (PMC9135694; doi:10.1038/s42003-022-03453-2)
Supplement: Supplementary file 1 — Peer Review File [file 42003_2022_3453_MOESM1_ESM.pdf]

nature portfolio

# Peer Review File

# The Pel polysaccharide is predominantly composed of a dimeric repeat of α-1,4 linked galactosamine and N-acetylgalactosamine.

**Open Access** This file is licensed under a Creative Commons Attribution 4.0 International License, which permits use, sharing, adaptation, distribution and reproduction in any medium or format, as long as you give appropriate credit to the original author(s) and the source, provide a link to the Creative Commons license, and indicate if changes were made. In the cases where the authors are anonymous, such as is the case for the reports of anonymous peer reviewers, author attribution should be to 'Anonymous Referee' followed by a clear attribution to the source work. The images or other third party material in this file are included in the article's Creative Commons license, unless indicated otherwise in a credit line to the material. If material is not included in the article's Creative Commons license and your intended use is not permitted by statutory regulation or exceeds the permitted use, you will need to obtain permission directly from the copyright holder. To view a copy of this license, visit http://creativecommons.org/licenses/by/4.0/.

Reviewers' comments:

Reviewer #1 (Remarks to the Author):

This manuscript by Sheppard and colleagues reports on the determination of the structure and anomeric configuration of Pel polysaccharide. A combination of mass spectrometry, NMR measurements, enzymatic digests, and enzymatic re-acetylation demonstrates that Pel consists of 1,4-linked galactosamine and N-acetylgalactosamine. Dimeric repeats of galactosamine and N-acetylgalactosamine are predominant in Pel, while poly-N-acetylgalactosamine oligomers and partially deacetylated oligomeric variants are also present. These observations contrast with prior work which suggested that Pel consists of GalNAc and GlcNAc. The absence of the latter in Pel extracts studied in this report is attributed to an improved purification process. This report is likely to be of interest to researchers working in the area of bacterial biofilms and, in my opinion, is appropriate in scope for this journal. There are, however, a few minor points that could be considered prior to publication.

1. Central to this study is an engineered strain of P. aeruginosa that overproduces the Pel polysaccharide. The authors may consider, and where relevant discuss, if the engineered pathway or overproduction may in any way bias the chemical composition of Pel in this strain when compared to Pel produced by unmodified bacterial strains.

2. The presentation of material in the manuscript tends to presuppose that the reader has significant knowledge of and appreciation for Pel polysaccharide. For a more general audience biology community, the manuscript could be strengthened by elaborating on the impact and significance of knowing the chemistry and anomeric structure of Pel. Some of this is alluded to in the final paragraph, but could be expanded upon, in my opinion.

Reviewer #2 (Remarks to the Author):

The manuscript entitled "The Pel polysaccharide is predominantly composed of a dimeric repeat of a-1,4 linked galactosamine and N-acetylgalactosamine" deals with the structural characterization of the biofilm matrix exopolysaccharide Pel, overproduced from a genetically engineered P. aeruginosa strain.

The manuscript suffers from critical flaws in the characterization section, in particular related to the NMR section, critical to assess the structure of P. auruginaoe Pel polysaccharide. The analysis of the NMR spectra needs to be more detailed, and some more experiments need to be acquired. Moreover, in my opinion the structure itself is not enough to justify publication on Communications biology, and once amended the manuscript can be published elsewhere.

Here some points:

Anomeric configuration, not conformation!

"our results reveal that Pel is a linear partially de-N-acetylated α-1,4-linked GalNAc polymer." Not clear how the authors derived this info from the NMR spectra. Have the authors acquired Heteronuclear 2D NMR spectra, did the authors attributed 13C and 1H resonances? The authors should provide a table with the NMR attribution. Chemical shifts are key to evaluate the polysaccharide structure.

"Anomery was confirmed by 2D 1H-1H Correlation Spectroscopy (COSY) spectra" not clear how the authors determined the anomeric configuration (anomery?) using a COSY in absolute. J coupling constants, homo- and hetero-nuclear are key to assess the anomeric configuration of sugar residues.

MALDI-MS spectra highlight the predominant present of GalN-GalNAc repeating units. Have the authors any indication of this repeating unit also from NMR data? There seem to be several

anomeric signals in the 1H spectrum, are they only due to oligoes with different lengths? have the authors try to separate them?

Figure 1b "reducing end mutarotation" please amend. The authors should explain why the intensity of the reducing a-anomers are (or looks) higher that the b-anomers.

Reviewer #3 (Remarks to the Author):

The Pel polysaccharide is predominantly composed of a dimeric repeat of α-1,4 linked
2 galactosamine and N-acetylgalactosamine.

In this manuscript, Le Mauff and Razvi et al. describe the isolation and characterization of the Pel polymer from Pseudomonas aeruginosa. Pel is an exopolysaccharide involved in biofilm formation in a number of different bacteria. To examine Pel structure, the authors overproduced the biopolymer in an engineered strain of P. aeruginosa, then purified the polymer, and characterized it through a combination of MS and NMR in conjunction with enzymatic digestion experiments. Through this work they show that Pel consists of a repeating motif of GalN-alpha-1,4-GalNAc. Overall, this work is of interest, provides clarification to the structure of Pel, and is reasonably well written, though at times the brevity of the writing leads to a lack of clarity.

The following points should be considered

Line 52: It may be useful to further detail the functions of the deletions used in the overproduction of Pel and/or to provide a reference as to the function of these or previous application of this strain.

Line 54: Could the authors clarify whether the GlcNAc referenced on line 54 is free GlcNAc or from a different polysaccharide or even low mwt Pel. ALSO…the authors should clarify that "The monosaccharide composition of Pel after hydrolysis was….

Figure 1a: The data points in Figure 1a are too small/indistinct for the reader to discern those for "PAO1 ΔwspF Δpsl Δpel" from those for "PAO1 ΔwspF Δpsl Δpel pBADpel". Likewise the colours/shading of the histogram are impossible to discern.
Data in Figure 1 b was confusing at first…until I realised that the polymer must have been almost completely hydrolysed…such that the average lengths must be MUCH less than that of the octasaccharide in 1c based upon the size of the reducing end anomeric proton peaks relative to those of the internal anomeric protons in the two cases. Either that or there is a lot of GalNAc in the sample. The authors should provide MS analysis of this same sample to provide independent information on what is being measured.

Figure 1f: The text in Figure 1F denoting m/z differences for HexNAc and HexN in the fragmentation profile are too small to be read at 100% zoom.

Figure 2: Inclusion of a MALDI-TOF spectra of native Pel that has been treated with a PBS control might aid in interpretation of the results.

Figure 2: It should be stated in the caption that panels g and h consist of Pel that has been re-N-acetylated before treatment with PBS or Agd3. As is, it is unclear what the treatment is and the panel configuration (with re-acetylated Pel on the right hand side) suggests that with the -ve control (PBS) the polymer is near completely N-acetylated naturally.

SI Figure 1: Annotation of the relevant peaks/couplings might make it clearer to the reader what the authors are aiming to show.

Line 124 state what reagent was used for TMS derivatisation
Line 126. What is methanolic acid? Is it HCl in methanol? If so…state this as chemists do not use

this term.
Line 130 hexamines should be hexosamines
Line 156 What is “anomery”

March 9th, 2022

Re: Manuscript revision "**The Pel polysaccharide is predominantly composed of a dimeric repeat of α-1,4 linked galactosamine and *N*-acetylgalactosamine.**"

Dear reviewers,

Kindly find below our answers (in red) to the reviewer's queries. We have uploaded a revised manuscript and look forward to hearing from you regarding this submission.

Sincerely

Don Sheppard, MD

**Response to reviewer comments:**

Reviewer #1:

1. Central to this study is an engineered strain of *P. aeruginosa* that overproduces the Pel polysaccharide. The authors may consider, and where relevant discuss, if the engineered pathway or overproduction may in any way bias the chemical composition of Pel in this strain when compared to Pel produced by unmodified bacterial strains.

A brief literature review comparing the Pel overproducing strain and the wildtype strain intrinsically producing Pel has been added to the discussion section of our article (starting on line 182).

2. The presentation of material in the manuscript tends to presuppose that the reader has significant knowledge of and appreciation for Pel polysaccharide. For a more general audience biology community, the manuscript could be strengthened by elaborating on the impact and significance of knowing the chemistry and anomeric structure of Pel. Some of this is alluded to in the final paragraph, but could be expanded upon, in my opinion.

The introduction has been expanded in the revised manuscript and now contains an introduction about the significance of *Pseudomonas aeruginosa* biofilms, exopolysaccharides, and Pel biosynthesis.

Reviewer #2:

Anomeric configuration, not conformation!

This error has been corrected. During the preparation of the revised manuscript, we also consulted with a polysaccharide NMR expert (Dr. Todd Lowary) to ensure the proper terminology was used throughout the manuscript. In recognition of this contribution and his helpful feedback regarding our NMR analysis and results section, we have added his name to the acknowledgments section. We trust that the revised manuscript now addresses your concerns.

"our results reveal that Pel is a linear partially de-N-acetylated α-1,4-linked GalNAc polymer."

Not clear how the authors derived this info from the NMR spectra. Have the authors acquired Heteronuclear 2D NMR spectra, did the authors attributed 13C and 1H resonances? The authors should provide a table with the NMR attribution. Chemical shifts are key to evaluate the polysaccharide structure.

The short communication format of our original manuscript led to confusion regarding our statements. We have paid particular attention to clarify this issue in the revised version. The reviewer is quite correct that the NMR experiments were not the sole source of information being summarized in this phrase.  The purpose of our NMR experiment was solely to investigate the anomeric configuration of the GalNAc within Pel. The experimental strategy employed was a comparative approach using a synthetic alpha-1,4-GalNAc octasaccharide and the oligosaccharides released from the treatment of re-*N*-acetylated Pel by treatment with its native glycoside hydrolase, $PelA_h$. Following expert advice, we now present a more detail explanation of our approach, the results, as well as peak assignments and have written a results section dedicated exclusively to the determination of anomeric configuration. The complete characterization of the polymer was made possible only by combining the results from our GC-MS, MALDI-TOF as well as NMR experiments.

"Anomery was confirmed by 2D 1H-1H Correlation Spectroscopy (COSY) spectra" not clear how the authors determined the anomeric configuration (anomery?) using a COSY in absolute. J coupling constants, homo- and hetero-nuclear are key to assess the anomeric configuration of sugar residues.

In the light of the comments received on the 2D NMR performed, and the lack of additional information it provides, we have elected to remove this study from the manuscript.

MALDI-MS spectra highlight the predominant present of GalN-GalNAc repeating units. Have the authors any indication of this repeating unit also from NMR data? There seem to be several anomeric signals in the 1H spectrum, are they only due to oligoes with different lengths? have the authors try to separate them?

In the NMR experiment, Pel was chemically re-*N*-acetylated prior to hydrolysis by $PelA_h$. As a result, the detection of repeating GalN-GalNAc units was not possible.

As the reviewer suggests, use of $PelA_h$ to obtain soluble material for NMR analysis resulted in the generation of Pel oligosaccharides with different lengths. The hydrolysis of the polymer is indeed the source of the multiple anomeric signals observed, and we have added a comment relating to the mutarotation we have seen previously with hydrolase treatment of the GAG polymer.

We have not tried to purify the individual oligomers, as previous attempts resulted in insufficient yield to perform the NMR experiment.

Figure 1b "reducing end mutarotation" please amend. The authors should explain why the intensity of the reducing a-anomers are (or looks) higher that the b-anomers.

The annotation has been removed from the figure for clarity, and the mutarotation event has been discussed more extensively in the results section (line 120).

Reviewer #3:

Line 52: It may be useful to further detail the functions of the deletions used in the

overproduction of Pel and/or to provide a reference as to the function of these or previous application of this strain.

A brief explanation of the mutations present in Pel-overproducing strain, and how this strain has been used for studies in the past has been added to the introduction.

Line 54: Could the authors clarify whether the GlcNAc referenced on line 54 is free GlcNAc or from a different polysaccharide or even low mwt Pel. ALSO…the authors should clarify that "The monosaccharide composition of Pel after hydrolysis was….

We believe that the GlcNAc observed in previous reports originated from contaminating peptidoglycan. The results section has been expanded to specify this information (starting on line 96).

Figure 1a: The data points in Figure 1a are too small/indistinct for the reader to discern those for "PAO1 ΔwspF Δpsl Δpel" from those for "PAO1 ΔwspF Δpsl Δpel pBADpel". Likewise the colours/shading of the histogram are impossible to discern.

The Figure has been redesigned to address these comments.

Data in Figure 1 b was confusing at first…until I realised that the polymer must have been almost completely hydrolysed…such that the average lengths must be MUCH less than that of the octasaccharide in 1c based upon the size of the reducing end anomeric proton peaks relative to those of the internal anomeric protons in the two cases. Either that or there is a lot of GalNAc in the sample. The authors should provide MS analysis of this same sample to provide independent information on what is being measured.

Unfortunately, this sample was not analyzed by mass spectrometry post NMR acquisition. However, our previous studies of $PelA_h$ showed that this enzyme could degrade substrate of a minimal size of 7 GalNAc, releasing products as small as GalNAc trimers (reference 24 of the manuscript). This previous mass spectrometry study also demonstrated the length heterogeneity of $PelA_h$ products. This heterogeneity can be modulated by the concentration of $PelA_h$ used, the concentration of substrate present, and incubation time. In the context of our NMR experiment, to maximize the amount of product which can be obtained and reduce this heterogeneity, we used a high concentration of $PelA_h$ and incubated it with Pel over a long period of time. We have added a short introduction of our experimental strategy to the results and discussion section for more clarity (line 106).

Figure 1f: The text in Figure 1F denoting m/z differences for HexNAc and HexN in the fragmentation profile are too small to be read at 100% zoom.

Thank you for this comment. The international nomenclature of monosaccharide representation has been chosen to replace the HexNAc and HexN in the Figure to improve clarity.

Figure 2: Inclusion of a MALDI-TOF spectra of native Pel that has been treated with a PBS control might aid in interpretation of the results.

This spectrum was acquired, however was devoid of any signal as, in the absence of any glycoside hydrolase treatment, no oligosaccharides were released from high molecular weight Pel, and therefore none were detected. This spectrum has been included as Supplemental Figure 2.

Figure 2: It should be stated in the caption that panels g and h consist of Pel that has been re-N-acetylated before treatment with PBS or Agd3. As is, it is unclear what the treatment is and the panel configuration (with re-acetylated Pel on the right hand side) suggests that with the -ve control (PBS) the polymer is near completely N-acetylated naturally.

These 2 spectra are now an independent figure (Figure 5). We have changed the legend as requested to address this comment.

SI Figure 1: Annotation of the relevant peaks/couplings might make it clearer to the reader what the authors are aiming to show.

In the light of the comments received on the 2D NMR performed, and the lack of additional information provided, we have elected to remove this study from the manuscript.

Line 124 state what reagent was used for TMS derivatization

The material and method section has been revised and now contains all pertinent information related to the experimental approach. (line 264: "Residues were then silylated with a mix of hexamethyldisiloxane : trimethylchlorosilane : pyridine (3:1:9).")

Line 126. What is methanolic acid? Is it HCl in methanol? If so…state this as chemists do not use this term.

We used HCl in methanol, a correction has been made to the material and method.

Line 130 hexamines should be hexosamines

This was corrected.

REVIEWERS' COMMENTS:

Reviewer #1 (Remarks to the Author):

The authors have satisfactorily addressed my critique of the initial submission in the revised version of the manuscript. It is my opinion that this work would be a welcomed addition to the literature.

Reviewer #2 (Remarks to the Author):

The authors have satisfactorily remodulated the paper. As for my concerns related to the NMR data, the authors well decided to reduce contribution of NMR analyses to the characterization of the polymers. This was a good choice since the way NMR is used is not sufficient to assess any structural/compositional information -

Reviewer #3 (Remarks to the Author):

The authors have adequately responded to my concerns. I recommend acceptance.
